# Editorial: New insights in veterinary cancer immunology

**DOI:** 10.3389/fvets.2024.1440527

**Published:** 2024-06-14

**Authors:** Carlos Eduardo Fonseca-Alves, Felisbina Luísa Queiroga, Cristina de Oliveira Massoco

**Affiliations:** ^1^Institute of Veterinary Oncology—IOVET, São Paulo, Brazil; ^2^Laboratory VetPrecision, Botucatu, Brazil; ^3^Animal and Veterinary Research Center (CECAV), University of Trás-os-Montes and Alto Douro, Vila Real, Portugal; ^4^Department of Veterinary Pathology, School of Veterinary Medicine and Animal Sciences, University of São Paulo—USP, São Paulo, Brazil

**Keywords:** dogs, immune system, immunotherapy, immuno-oncology, tumor-associated inflammation

## 1 Introduction

Veterinary cancer immunology is an emerging area that has been growing in the past years and is focused on understanding how the immune system interacts with animal cancer cells (1). In humans, immunology is one of the most studied areas in cancer research with new technologies being created for patient treatment. One of the most notable discoveries in recent years was the creation of a CAR-T cell therapy for hematopoietic cancer treatment (2). Cancer immunology explores the mechanisms by which the immune system can detect and destroy cancer cells, as well as how tumors evade immune surveillance (3). Research in veterinary cancer immunology has revealed that, similar to humans, animals' immune systems can recognize cancer-specific antigens and mount a response (1, 4). However, tumors often develop strategies to suppress the immune response, such as producing immunosuppressive cytokines or recruiting regulatory T cells that inhibit anti-tumor activity. Understanding these interactions is crucial for developing effective immunotherapies for animal cancers (5).

Recent advancements in veterinary cancer immunology include the development of novel immunotherapeutic approaches. These include cancer vaccines that stimulate the immune system to recognize and attack tumor cells, monoclonal antibodies that target specific cancer antigens, and checkpoint inhibitors that block proteins used by cancer cells to evade immune detection (4, 6, 7). Clinical trials in veterinary medicine have shown promising results, particularly in treating canine cancers like lymphoma (4) and osteosarcoma (8). These therapies not only improve the prognosis and quality of life for animals but also provide valuable insights that can translate to human oncology.

Another important aspect of veterinary cancer immunology is the study of comparative oncology, which examines cancer across different species to identify common mechanisms and potential treatments (9). Animals, particularly dogs, develop cancers that are biologically similar to human cancers, making them excellent models for research. By studying cancer in animals, researchers can gain a better understanding of tumor biology, immune response, and the efficacy of new treatments. This comparative approach helps bridge the gap between veterinary and human oncology, leading to advancements that benefit both animal and human patients (10).

In recent years, significant progress has been made in understanding the relationship between the immune system, inflammation, and various cancer subtypes. For this reason, the Research Topic “*New insights in veterinary cancer immunology*” focused on the recent advances in veterinary cancer immunology and compilated original and review articles that provide the most recent advances in veterinary cancer immunology.

Lymphomas in dogs can be categorized based on their cellular origin (B-cells or T-cells) using specific surface markers like CD21/CD79a for B-cells and CD3/CD4/CD8 for T-cells. Bae et al. conducted an interesting investigation to examine the clinical implications of aberrant lymphoma phenotypes in canine patients. For this, 27 dogs with aberrant lymphoma diagnosed through flow cytometry were analyzed for paraneoplastic syndromes, prognostic factors, and clinical features. Common aberrancies included the immunophenotype MHCII- and CD3^+^/CD21^+.^ B-cell lymphomas frequently exhibited MHCII^−^, CD3^+^/CD21^+^, CD34^+^, and CD79a^−^, while T-cell lymphomas showed CD3^+^/CD21^+^, CD4^−^/CD8^−^, CD5^−^, and CD45^−^. The platelet-neutrophil ratio and serum albumin levels varied significantly among different immunophenotypes. CD34 expression correlated with cranial mediastinal mass, clinical substage, and fever, while MHCII expression was linked to chemotherapy reactions and fever. Despite these correlations, no significant differences in survival periods were observed among the phenotypic aberrancies. The study concludes that aberrant lymphomas are common in dogs, with specific clinical prognostic factors linked to these immunophenotypes.

Altamura and Borzacchiello published a valuable opinion article regarding feline oral papillomavirus-inducing oral squamous cell carcinoma in cats in comparison with human Head and neck squamous cell carcinoma (HNSCC). In humans, HNSCC is a prevalent cancer affecting the aerodigestive tract in humans, with tobacco, alcohol, and poor oral hygiene as primary risk factors. However, around 25% of these cases, particularly oral squamous cell carcinoma (OSCC), are linked to high-risk human papillomavirus (HPV), mainly HPV-16 and HPV-18. These HPV-associated cancers typically occur in the oropharynx, with viral oncogenes E6 and E7 disrupting tumor suppressors like p53 and pRb. Similarly, in veterinary medicine, papillomaviruses (PVs) are implicated in squamous cell carcinomas (SCC) in cattle and horses, and there is growing evidence of their role in feline OSCC (FOSCC), with various studies showing significant associations between feline PVs (FcaPVs) and FOSCC. In this manuscript, the authors described those initial studies identified FcaPV-2 in FOSCC, revealing its transforming properties similar to those of HPV in human cancers. Subsequent research confirmed the presence of FcaPV-2 in FOSCC-derived cell lines and actual tumor samples, with high viral loads and active viral gene expression linked to oncogenic processes. Studies from different regions, including Italy, Japan, and Germany, have consistently detected FcaPV-2 in FOSCC, reinforcing the virus's role in tumor development. Despite the variability in reported association rates, ranging from 5 to 58%, the evidence points to a significant co-causative role of FcaPVs in FOSCC, warranting further research and consideration in veterinary oncology. In conclusion, the association between FcaPVs and FOSCC highlights the need for additional studies to understand this relationship better and potentially reclassify FcaPV-positive FOSCC as a distinct clinical entity. This could lead to tailored diagnostic and therapeutic approaches, similar to HPV-related HNSCC in humans. Future research should focus on multicentric studies to monitor these tumors' biological behavior and response to treatments, ultimately improving feline patients' diagnosis, treatment, and prognosis.

Rodney et al. published a pioneer study that investigated the genomic landscape and alteration of gene expression in cats with feline oral squamous cell carcinoma (FOSCC). FOSCC is a prevalent and aggressive cancer in cats, accounting for up to 80% of feline oral cancers and typically having a poor prognosis. Using whole exome sequencing (WES) and RNA sequencing, these researchers have identified somatic mutations and gene expression changes linked to FOSCC. This study is the first to apply WES to feline cancer and revealed tumor-associated mutations in six cats, with notable similarities to mutations found in human head and neck squamous cell carcinoma (HNSCC). Mutations in the TP53 gene, common in many cancers, were found in four samples, each with unique mutations. Other mutations were discovered in genes related to cellular growth control, such as KAT2B and ARID1A. Gene expression analysis indicated molecular similarities to human oral squamous cell carcinoma (OSCC), including changes in pathways related to epithelial to mesenchymal transition and the IL6/JAK/STAT pathway. These findings enhance the understanding of FOSCC and support its use as a comparative model for studying human HNSCC.

Chimeric antigen receptors (CARs) have been used in human oncology and have shown exceptional potential for patient treatment. Over the last 20 years, the investigation of CARs has advanced precipitously in recent decades as a state-of-the-art treatment option for several cancer subtypes. However, the use of this technique in Veterinary Oncology remains under development and clinical trials have only recently been initiated in dogs. Cockey and Leifer contributed to this Research Topic with a relevant mini-review manuscript, highlighting the potential of CARs in veterinary immuno-oncology. CARs are artificially designed proteins that combine a specific tumor associated antigen-binding single-chain variable fragment with the signaling domain of a T cell receptor and its co-receptors (TCR signaling complex). T cells from patients are engineered to express a CAR, enabling them to identify and destroy target cells, typically hematological cancers. While the U.S. Food and Drug Administration (FDA) has approved several CAR T-cell therapies for human use, there are numerous obstacles to adapting these treatments for veterinary use. This review addresses key considerations for using CARs in veterinary settings, including CAR design and choice of cell carriers, and explores the potential future of CAR therapy in veterinary oncology. This manuscript brings a critical approach to CARs in veterinary oncology and provides new insights for future studies.

The tumor microenvironment (TME) plays a critical role in cancer development, progression, and response to immunotherapy. It significantly influences cancer cell behavior and can contribute to tumor aggressiveness. Recognizing the importance of TME proteins, Brambilla et al. conducted a relevant study that investigated the role of Periostin (POSTN) in canine patients with bladder urothelial carcinoma (BUC). POSTN is an extracellular matrix protein involved in tissue regeneration and metastasis. In humans, high POSTN levels are linked to aggressive tumors and poor prognosis in various cancers, except for BUC, where POSTN is downregulated. Since the role of POSTN is unclear in veterinary medicine, these authors examined POSTN expression in canine BUCs, using 45 tumor samples and 6 non-neoplastic bladder samples. Results showed that POSTN expression was generally lower in tumors compared to normal tissue, with a significant inverse correlation between POSTN expression and mitotic count. These findings suggest that POSTN could be a prognostic marker and a potential target for anticancer therapies in canine BUCs.

The transformation from adenoma to carcinoma in human colorectal cancer (CRC) is well-known, but its transcriptomic features in canines remain unclear. Lin et al. assessed for the first time in veterinary medicine, the transcriptome data from normal, adenoma, and cancerous canine colon tissues to investigate a malignant progression. These authors identified several genomic alterations with differential analysis highlighting the role of PFKFB3 in this transformation. Enrichment analysis showed metabolic dysregulation, immunosuppression, and typical cancer pathways in canine colorectal tumors. Dynamic expression patterns of differentially expressed genes (DEGs) and network analysis revealed inflammatory and immune pathways' roles, with the S100A protein family involved in malignancy. They identified five novel CRC markers, with GTBP4 showing strong diagnostic and prognostic potential. This study provides new biomarkers and comparative evidence for CRC research in humans and dogs.

Klingemann, wrote a review article about the use of *Viscum album* in Veterinary Oncology. *Viscum album*, commonly known as mistletoe, is a plant with notable medicinal properties. This paper explores the potential of *Viscum album* L. (mistletoe) extract, which has been used in human cancer treatment, for canine cancer therapy. It contains compounds like lectins and viscotoxins, which have anti-cancer and immune-boosting effects. Additionally, the extract's lectin ML-1 can enhance patient wellbeing by triggering endorphin release. Given its cross-reactivity with canine cells and low side effect profile, mistletoe extract may be a viable treatment option for dogs with cancer. In oncology, mistletoe extract is used to inhibit cancer cell proliferation and improve patient wellbeing by stimulating endorphin release. Its therapeutic potential extends to both human and veterinary medicine due to its relatively low side-effect profile.

Understanding a tumor's immune context is crucial in cancer research. Canine oral melanoma serves as a good model for human immunotherapy, but more studies are needed to understand its immune landscape and its similarity to human melanoma. Vanhaezebrouck et al. performed a retrospective study analyzing RNA sequences from formalin-fixed paraffin-embedded (FFPE) tissue samples of canine oral melanomas using Nanostring Technology. The relevant study compared melanoma tumors restricted to the oral cavity (OL) and primary oral tumors with a history of metastasis (OM). Normal buccal mucosa samples were used as a reference. The OM group showed significant gene expression changes compared to the OL group, including decreased expression of genes like S100, BRAF, and BCL2, and increased expression of hypoxia-related genes (VEGFA), cell mobility genes (MCAM), and PTGS2 (COX2). Immune landscape analysis indicated a shift in the OM group from a potentially active “hot” tumor suppressed by immune checkpoints to heightened expression of checkpoints and immune blockades like PD1 and IDO2. The OM group also had reduced expression of Toll-like receptors (TLR4) and IL-18, aiding in immune escape, and signs of immune cell exhaustion were evident in both groups through increased TIGIT expression. Both groups showed higher CD4 expression compared to normal tissue, but CD4 expression decreased significantly in OM tumors compared to OL. This preliminary study highlights significant gene expression differences between localized and metastatic canine oral melanomas.

Razmara et al. wrote a pertinent review article focused on the role of natural killer (NK) cells in canine cancer immunotherapy. The field of cancer immunology has gained significant attention due to immunotherapies that effectively target immune cells, achieving notable anti-tumor effects. Despite this progress, successful cellular immunotherapy for solid tumors remains difficult. Dogs with naturally occurring cancers provide a valuable model to address these challenges, especially for NK cell-based therapies. This review highlights recent advancements in understanding canine NK cells, crucial for future translational NK immunotherapy research given their innate cytotoxicity against a broad array of targets and their potent cytokine production. Characterizing canine NK cells is important due to the challenges in defining them and the limited availability of specific reagents. The review also summarizes current clinical and translational studies using NK cell immunotherapy in canine cancer patients. These studies offer insights into efficacy and immune responses, paving the way for novel combinational therapies. This knowledge enhances the canine cancer model and supports translational efforts to improve cancer treatments for both dogs and humans.

Summing up, all the manuscripts published in this Research Topic offer a fresh insight into the use of the immune system for various therapeutic strategies in veterinary oncology. These advancements have the potential to broaden the adoption of immunotherapies in clinical practice and pave the way for more translational comparative studies. Ongoing research into immunotherapies approaches in veterinary oncology remains critical to refine cancer management and treatment options for canine patients.

## Author contributions

CF-A: Conceptualization, Data curation, Formal analysis, Funding acquisition, Investigation, Methodology, Project administration, Resources, Software, Supervision, Validation, Visualization, Writing – original draft, Writing – review & editing. FQ: Conceptualization, Data curation, Formal analysis, Funding acquisition, Investigation, Methodology, Project administration, Resources, Software, Supervision, Validation, Visualization, Writing – original draft, Writing – review & editing. CM: Conceptualization, Data curation, Formal analysis, Funding acquisition, Investigation, Methodology, Project administration, Resources, Software, Supervision, Validation, Visualization, Writing – original draft, Writing – review & editing.
